# Endoscopic treatment for duodenal perforation due to biliary stent dislocation: A case report and brief review of the literature

**DOI:** 10.1097/MD.0000000000031868

**Published:** 2022-12-02

**Authors:** Yuki Fujii, Kazuyuki Matsumoto, Kazuya Miyamoto, Akihiro Matsumi, Kosaku Morimoto, Hiroyuki Terasawa, Tatsuhiro Yamazaki, Shigeru Horiguchi, Koichiro Tsutsumi, Hironari Kato

**Affiliations:** a Department of Gastroenterology and Hepatology, Okayama University Graduate School of Medicine, Dentistry and Pharmaceutical Science, Okayama, Japan.

**Keywords:** biliary stent, endoscope, migration, perforation

## Abstract

Duodenal wall perforation by a dislocated biliary stent placed for biliary structure is rare but can be life-threatening. There are few reports on the management of stent-related duodenal perforation.

**Patient concerns:**

Three cases included in this study had undergone endoscopic retrograde cholangiopancreatography with placement of a plastic stent for biliary stricture. Two cases had symptoms (fever or abdominal pain), while other case showed no symptom after biliary stent placement.

**Diagnoses:**

Dislocation of plastic stents was revealed on computed tomography or endoscopic images. Two patients were diagnosed with duodenal perforation due to distal migration of long stents with a straight shape on the distal side. One patient was diagnosed with fistula formation between the intrahepatic bile duct and duodenum due to perforation of a pigtail stent.

**Interventions:**

All cases could successfully be managed endoscopically with closure by hemoclips or stent replacement.

**Outcomes:**

All 3 cases were improved after endoscopic treatment without any subsequent intervention.

**Lessons:**

Longer stents with a straight distal side are associated with a higher risk of duodenal perforation. Endoscopic management is appropriate as a first-line approach for a clinically stable patient. At the time of stent placement, we should pay attention to the length and type of stent.

## 1. Introduction

Endoscopic biliary drainage using a plastic stent (PS) is frequently performed during endoscopic retrograde cholangiopancreatography (ERCP) to resolve malignant and benign biliary obstructions. PS migration after ERCP occurs in approximately 5% of cases and in very rare cases (incidence: <0.1%) may lead to intestinal perforation.^[1]^ Intestinal perforation is a rare but potentially life-threatening adverse event associated with stent placement. Perforation due to biliary stent dislocation can occur in the duodenum, jejunum, ileum, cecum, and colon.^[2]^ Once perforation has occurred, an early diagnosis and management – including endoscopic and surgical closure of the perforation site – are essential. The clinical presentation can sometimes be asymptomatic and nonspecific with symptoms that mimic those of cholecystitis or pancreatitis, thereby obscuring early detection.^[3]^ As a result, a high index of suspicion is required to diagnose this complication. We experienced 3 successfully treated cases of duodenal perforation caused by a biliary stent. We herein present these cases with typical endoscopic and radiographic images and discuss the cause and treatment method with a review of the relevant literature. Written informed consent was obtained from patients or legal guardians for publication of the case report. Ethnical approval was not necessary in our hospital, because this case report evaluated less than 10 patients.

## 2. Case report

Cases managed in our center between January 1, 2012 and June 30, 2022 were obtained from the prospectively maintained ERCP database. Out of 2898 ERCPs in which biliary PSs were placed during this period, 3 cases (0.10%) of duodenal perforation caused by PS displacement were identified. Individual data of the screened cases, including symptoms, stenting indication, characteristics of the dislocated stent, time from stent placement to perforation, treatment, and outcome were extracted. The endoscopically-treated cases are summarized in Table 1.

**Table 1:** Summary of endoscopically-treated cases of duodenal perforation due to dislocation of a biliary tract stent.

Case no.	Age/sex	Diagnosis	Chief complaint	Diagnostic method	Interval from stent insertion to perforatin (days)	Location of stricture	Placed stent diameter/length(type)	Treatment	Outcome
1	72/F	Pancreatic cancer	Fever	CT	30	CBD and CD	BD:10mm/6cm (FCSEMS) GB:7Fr/28cm (cutting ENBD tube)	Removal of PS and closure of perforated wall with hemoclips	Improved
2	77/F	Mutliple liver cysts	Abdominal pain	CT	2	IHBD	B2:5Fr/28cm (cutting ENBD tube) B4:6Fr/25cm (cutting ENBD tube)	Removal of PS and closure of perforated wall with hemoclips	Improved
3	59/M	Duct to duct anastomosis with LDLT	None	Endoscopy	88	BA	B5:7Fr/6cm (Pigtail) B8: 7Fr/10cm (Straight)	Removal of PS and replacement with another PS	Improved

BA = biliary anastomosis, BD = bile duct, CBD = common bile duct, CD = cystic duct, CT = computed tomography, ENBD = endoscopic nasobiliary drainage, F = female, FCSEMS = fully covered self-expandable metalic stent, GB = gallbladder, IHBD = intrahepatic bile duct, LDLT = living donor liver transplantation, M = male, PS = plastic stent.

### 2.1. Case 1

**Figure 1.**
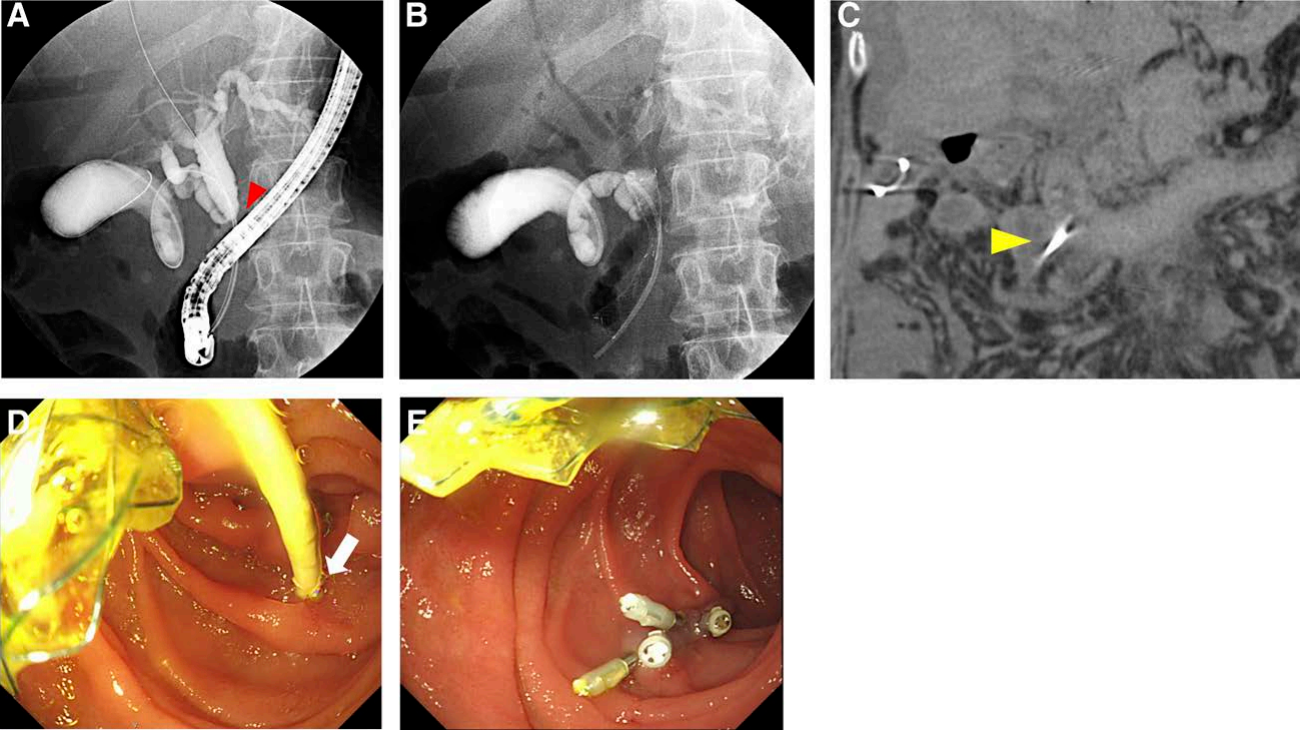
(A): ERCP revealed obstruction of the union of the cystic duct and the common bile duct (red arrowhead). (B): A 10 mm × 6 cm fully covered self-expandable metallic stent in the bile duct and an ENBD tube cut to 28 cm in the gallbladder to prevent cholecystitis were placed. (C): Abdominal CT revealed that the distal end of the gallbladder PS was protruding into the posterior peritoneum (yellow arrowhead). (D): The distal tip of the gallbladder stent penetrated the duodenal wall, which was confirmed during endoscopy (white arrow). (E): The perforation was closed using hemoclips. CT = computed tomography, ENBD = endoscopic nasal biliary drainage, ERCP = endoscopic retrograde cholangiopancreatography, PS = plastic stent.

A 72-years-old female patient suffering from jaundice with abdominal pain was diagnosed with unresectable pancreatic cancer with cholecystitis. ERCP revealed obstruction of the union of the cystic duct and common bile duct (Fig. 1a); thus, a 10 mm × 6 cm fully covered self-expandable metallic stent (BONASTENT, Medico’s Hirata Inc., Japan) was placed in the bile duct and a endoscopic nasal biliary drainage (ENBD) tube cut to a total stent length of 28 cm (nasal biliary drainage, Cook Medical Japan) was placed in the gallbladder (Fig. 1b). The patient’s symptoms improved and the patient discharged 10 days after ERCP. However, 1 month later, the patient complained of fever. Computed tomography revealed that the distal end of the gallbladder PS was protruding into the posterior peritoneum (Fig. 1c). We performed endoscopy and removed the PS using a forceps with closure of the perforated site using hemoclips (Fig. 1d, e). The patient was discharged on day 19 after the procedure.

### 2.2. Case 2

**Figure 2.**
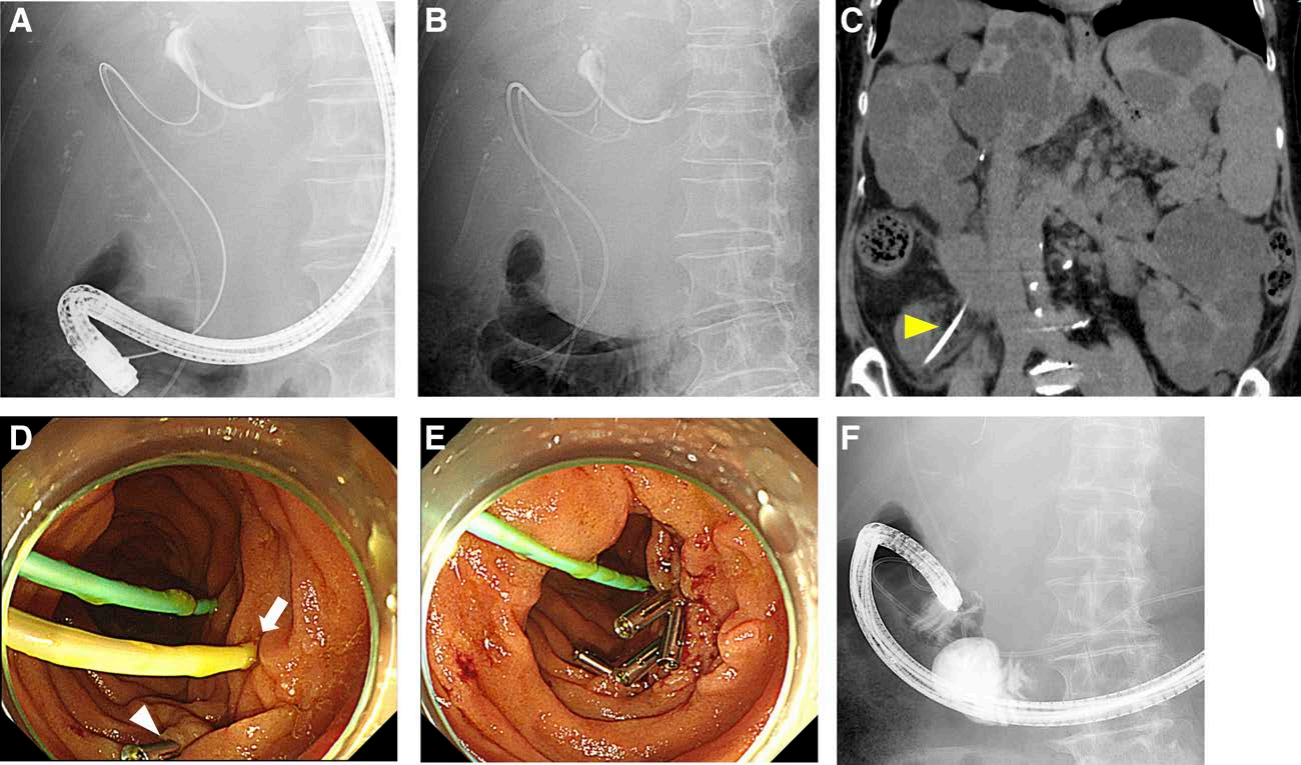
(A): The patient underwent ERCP due to stenosis of the intrahepatic bile duct caused by multiple liver cysts. (B): The patient underwent biliary drainage using a 5 Fr ENBD tube cut to 28 cm for B5 and a 6 Fr ENBD tube cut to 25 cm for B4. (C): CT revealed distal migration of the PS for B4 and duodenal perforation (yellow arrowhead). (D): The distal tip of the biliary stent penetrated the duodenal wall (white arrow). This was confirmed during endoscopy after marking the perforation site with hemoclips (white arrowhead). (E): The ENBD tube was removed using a forceps, and the perforated duodenal wall was closed using hemoclips. (F): Contrast injection after closure of perforation site showed no residual leakage. CT = computed tomography, ENBD = endoscopic nasal biliary drainage, ERCP = endoscopic retrograde cholangiopancreatography, PS = plastic stent.

A 72-years-old female patient with autosomal dominant polycystic kidney disease had repeatedly undergone ERCP for 6 years due to stenosis of the intrahepatic bile duct caused by multiple liver cysts. She underwent biliary drainage using a 5 Fr ENBD tube cut to a total stent length of 28 cm (nasal biliary drainage, Cook Medical Japan) for B2 and a 6 Fr ENBD tube cut to a total stent length of 25 cm (nasal biliary drainage, Cook Medical Japan) for B4 (Fig. 2a, b). After 2 days, she complained of right upper pain with tenderness. Computed tomography revealed distal migration of the PS placed for *B*4 and duodenal perforation (Fig. 2c). The ENBD tube was removed using a forceps and the perforated duodenal wall was closed using hemoclips (Fig. 2d, e). Contrast injection after closure of the perforation site did not show any residual leakage (Fig. 2f). The patient was discharged on day 12 after the procedure.

### 2.3. Case 3

**Figure 3.**
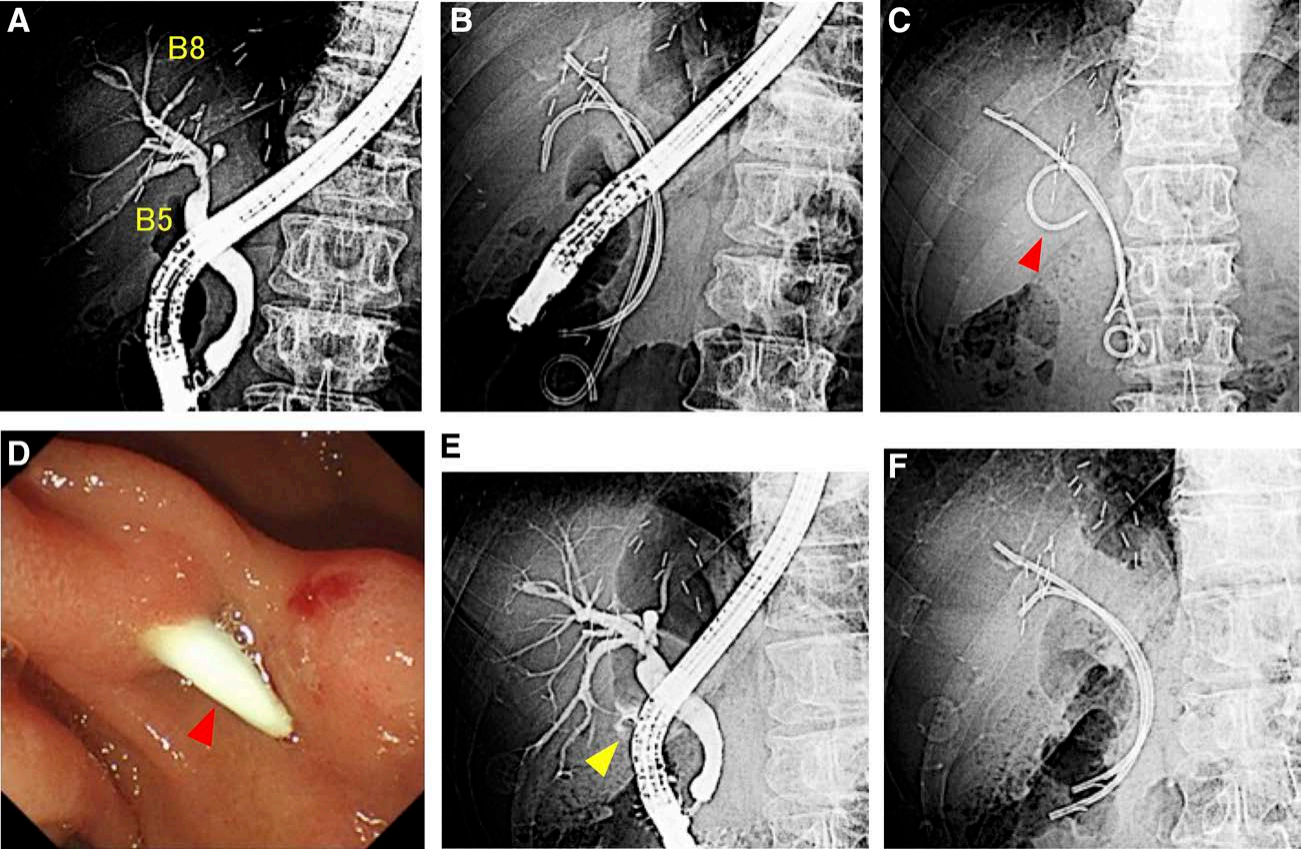
(A): ERCP showed anastomotic biliary stricture. (B): The patient underwent endoscopic biliary drainage using a pigtail stent for B5 and a straight stent for B8. (C): After 3 months, fluoroscopy before ERCP showing dislocation of the proximal tip of the biliary stent (red arrowhead). (D): The proximal tip of the biliary stent penetrated the duodenal wall. This was confirmed during endoscopy (red arrowhead). (E): ERCP showed contrast leakage from B5 (yellow arrowhead). (F): The straight stents of B5 and B8 were replaced. Three months later, ERCP showed no contrast leakage from B5. ERCP = endoscopic retrograde cholangiopancreatography.

The patient was a 59-years-old man with a history of living donor liver transplantation for liver cirrhosis, and hepatocellular carcinoma complicated by biliary anastomotic stricture after living donor liver transplantation. After repeated ERCP with balloon dilation and PS placement, he underwent endoscopic biliary drainage using a 7 Fr × 6 cm pigtail PS (Medi-Globe, Medico’s Hirata, Japan) for *B*5 and a 7 Fr × 10 cm straight PS (Throughpass, Gadelius Medical K.K., Japan) for *B*8 (Fig. 3a, b). The patient, who had been asymptomatic, was admitted 3 months later for the reevaluation of stricture. At ERCP, fluoroscopy showed dislocation of the proximal tip of the PS that had been placed in *B*5 (Fig. 3c). The endoscopic view revealed that the proximal tip of the PS had penetrated the duodenal wall through the liver parenchyma (Fig. 3d). ERCP showed contrast leakage from *B*5 (Fig. 3e), and the 7 Fr × 10 cm straight PS (Flexima™, Boston Scientific Co., Natick, MA) for *B*5 and 7 Fr × 12 cm straight PS (Flexima™, Boston Scientific Co., Natick, MA) for B8 were replaced (Fig. 3f). The patient had been asymptomatic and was discharged 2 days after ERCP. Three months later, ERCP showed no contrast leakage from *B*5 and the improvement of anastomotic stricture. None of the PSs required replacement.

## 3. Discussion

We report 3 cases of duodenal perforation due to PS dislocation in which endoscopic management was successfully. We reviewed the relevant literature listed in the PubMed database between 2000 and 2022, and identified 21 cases of stent-related duodenal perforation that were managed with endoscopic removal of the PS (Table 2).^[1,4–16]^

**Table 2:** Summary of cases of endoscopic removal of plastic stents found in the relevant literature.

Year	Sex/Age	Diagnosis	Chief complaint	Diagnostic method	Interval from stent insertion to perforatin	Dislocated Stent Diameter/Length/Type	Treatment	Outcome
2000 ^[4]^	74/M	HCCA	Abdominal pain and vomiting	Fluoroscopy	1 d	10Fr/15cm/straight	Closure of perforation site by hemoclip	Improved
2003 ^[5]^	86/M	HCCA	Jaundice	Fluoroscopy	20 d	10Fr/15cm/straight	Only endoscopic removal of PS	Died (8 d after endoscopy)
2011 ^[6]^	70/M	Benign bile duct stricture	Jaundice	CT	2 yr	8.5Fr/N.D./straight	Closure of perforation site by hemoclip	Improved
2012 ^[7]^	87/F	Choledocholithisis	Back pain	CT	a few d	8.5Fr/15cm/straight	Only endoscopic removal of PS	Died (9 d after endoscopy)
2012 ^[8]^	73/F	Cholangiocarcinoma	Fever	Endoscope	N.D.	N.D./N.D./straight	Closure of perforation site by hemoclip	Improved
2015 ^[9]^	48/F	Benign biliary stenosis	Abdominal pain and fever	CT	2 wk	N.D./N.D./straight	Closure of perforation site by OTSC	Improved
2016 ^[10]^	72/M	HCCA	Abdominal pain and fever	CT	1 mo	8.5Fr/12cm/straight	Closure of perforation site by OTSC	Improved
2016 ^[11]^	71/M	N.D.	N.D.	CT	N.D.	N.D./N.D./straight	Closure of perforation site by OTSC	Improved
2018 ^[12]^	79/F	N.D.	Abdominal pain	CT	N.D.	7Fr/12cm/straight	Closure of perforation site by hemoclip	Improved
2018 ^[13]^	57/M	PSC	Fever	Endoscope	2 mo	8.5Fr/15cm/straight	Closure of perforation site by hemoclip	Improved
2019 ^[14]^	71/F	Cholangiocarcinoma	Abdominal pain and fever	CT	1 d	8.5Fr/12cm/straight	Closure of perforation site by OTSC	Improved
2019 ^[15]^	84/F	Choledocholithisis	None	CT	2 mo	8.5Fr/10cm/straight	Closure of perforation site by hemoclip	Improved
2019 ^[15]^	93/F	Choledocholithisis	None	Endoscope	5 mo	8.5Fr/9cm/straight	Closure of perforation site by hemoclip	Improved
2019 ^[16]^	50/M	Biliary leak after laparoscopic cholecystectomy	Abdominal pain	CT	28 d	10Fr/10cm/straight	Closure of perforation site by hemoclip	Improved
2019 ^[16]^	78/F	GB cancer	Abdominal pain and fever	CT	3 d	7Fr/12cm/straight	Closure of perforation site by hemoclip	Improved
2019 ^[16]^	72/M	HCCA	Abdominal pain	CT	2 d	10Fr/12cm/straight	Closure of perforation site by hemoclip and fibrin glue	Improved
2019 ^[16]^	84/F	HCCA	Abdominal pain	CT	21 d	10Fr/12cm/straight	Closure of perforation site by hemoclip	Improved
2019 ^[16]^	73/F	GB cancer	Abdominal pain and janudice	CT	51 d	10Fr/15cm/straight	Closure of perforation site by hemoclip	Improved
2020 ^[1]^	72/M	Choledocholithisis	Abdominal pain	CT	33 d	8.5F/9cm/straight	Only endoscopic removal of PS	Improved
2020 ^[1]^	84/M	N.D.	Abdominal pain	CT	3 d	7Fr/12cm/straight	Closure of perforation site by hemoclip with NBD	Improved
2020 ^[1]^	52/M	Choledocholithisis	Abdominal pain and fever	CT	75 d	8.5Fr/9cm/straight	Closure of perforation site by hemoclip with NBD	Improved

CT = computed tomography, F = female, GB = gallbladder, HCCA = hilar cholangiocarcinoma, M = male, NBD = nasobiliary drainage, N.D. = not described, OTSC = over the scope clip, PS = plastic stent, PSC = primary sclerosing cholangitis.

Regarding symptoms, among these 21 perforated cases, 19 (90%) patients showed some symptoms, while 2 (10%) showed no symptoms. Abdominal pain or fever were common symptoms that were observed in 15 (71%) cases. The interval from stent insertion to perforation ranged from 1 day to 2 years, which demonstrated that stent-related duodenal perforation can occur regardless of stent duration.^[1,4–16]^ Six of the cases in the literature (29%) were diagnosed within 1 week after PS placement. Symptoms in such cases may mimic common complications of ERCP, such as post-ERCP pancreatitis, cholangitis or cholecystitis. When encountering patients with such symptoms, a high index of suspicion of the possibility of duodenal perforation is warranted and the diagnostic workup often requires cross-sectional imaging. In asymptomatic cases, including our 1 case, the interval from stent insertion to the diagnosis of perforation ranged from 2 to 5 months; thus, inserted stents might have been completely obstructed before migration, so bile juice was not evacuated into the abdominal cavity.

In the past reports,^[1,4–16]^ all of the stent-related duodenal perforations were caused by the distal migration of long straight biliary PSs, which are more prone to distal migration in comparison to pigtail PSs. In our case series, distal migration occurred in 2 cases and dislocation of the proximal tip of the PS occurred in 1 case. In 2 cases with distal migration, the PSs were longer than 20 cm with a straight shape on the distal side. We sometimes used a cut ENBD tube for peripheral bile duct or gallbladder drainage because there were no appropriate ready-made PSs of such a long length, especially for gallbladder drainage. After drainage of the gallbladder, straightening of the looped stent that was placed in the gallbladder caused duodenal perforation in one of our cases. These cases highlight that the use of a longer distal straight-type stent is associated with a higher risk of duodenal perforation. Stassen et al reported that stent length > 12 cm, perihilar stricture, and stent insertion into the left liver lobe were risk factors for stent migration-induced perforation of the duodenal wall.^[3]^ Although pigtail stents may be less traumatic than straight stents, as they are associated with a low rate of stent migration, we experienced duodenal perforation caused by dislocation of the proximal tip of a pigtail PS. Mismatch between the shapes of the bile duct and PS rarely caused dislocation of the proximal tip of a biliary PS. At the time of stent placement, we should pay attention to the shape and length of the stent, in accordance with the shape of the bile duct. To the best of our knowledge, there are no previous reports of duodenal perforation caused by migration of a gallbladder stent and dislocation of the proximal tip of a biliary stent.

Presently, no relevant guidelines exist for preventing and managing duodenal perforation secondary to stent dislocation. Among 21 perforated cases, 14 cases were treated endoscopically with hemoclips and 4 cases were treated with over-the-scope clips (OTSC). Three cases were treated with PS removal alone; however 2 of 3 patients died after endoscopy. Abdominal infection sometimes leads to death,^[2]^ and PS removal alone is not a suitable treatment for stent perforation. Surgical intervention should be considered in cases with failure of endoscopic therapy and/or persistent abdominal infection.^[3]^ Regarding closure methods, using an OTSC is better than using hemoclips; however, OTSCs are more expensive than hemoclips and the procedure is somewhat complicated. When the perforated hole caused by plastic stent migration is small, hemoclips are sufficient for closing the hole. Closure with OTSCs would be a treatment option after failed closure using hemoclips.

Duodenal perforation with PS dislocation is rare; however, we should consider the possibility of stent-related duodenal perforation after stent placement. Endoscopic retrieval of dislocated biliary stents with closure of the site of duodenal perforation is feasible in clinically stable patients. Longer straight-type stents are associated with a higher duodenal perforation risk; thus, we should pay attention that the shape and length of the stent are in accordance with the biliary tract shape.

## Author contributions

**Conceptualization:** Yuki Fujii, Kazuyuki Matsumoto.

**Investigation:** Kazuya Miyamoto, Akihiro Matsumi, Kosaku Morimoto, Hiroyuki Terasawa, Tatsuhiro Yamazaki, Shigeru Horiguchi, Koichiro Tsutsumi.

**Writing – original draft:** Yuki Fujii.

**Writing – review & editing:** Kazuyuki Matsumoto, Hironari Kato.
